# Contrasting the influences of phosphate and phosphite on growth of *Aspergillus niger*

**DOI:** 10.3389/fmicb.2025.1664730

**Published:** 2025-08-14

**Authors:** Ying Wang, Kejin Ding, Jiakai Ji, Meiyue Xu, Shihui Yan, Yonghong Fan, Dan Yu, Zhen Li

**Affiliations:** ^1^College of Resources and Environmental Sciences, Nanjing Agricultural University, Nanjing, Jiangsu, China; ^2^North China Power Engineering Co., Ltd. of China Power Engineering Consulting Group, Beijing, China; ^3^Jiangsu Provincial Key Lab for Organic Solid Waste Utilization, Nanjing Agricultural University, Nanjing, China

**Keywords:** phosphite, phosphate, reduced phosphorus material, *Aspergillus niger*, inhibitory effect

## Abstract

Phosphite serves as an alternative phosphorus material in terrestrial ecosystems. *Aspergillus niger* (*A. niger*), a prominent phosphate-solubilizing fungus (PSF), facilitates phosphorus and leaches heavy metal ions via organic acids and enzymes. With the synergistic effect of phosphate materials, heavy metal ions can be effectively immobilized by *A. niger* to achieve remediation of contaminated soils. This study investigated the structural distinctions between phosphite and phosphate compounds by using ATR-IR and Raman spectroscopy, while concurrently assessing the physiological impact of phosphite on *A. niger*. After incubation with phosphite, the average fungal biomass and acid phosphatase activities were reduced by approximately 50% with respect to phosphate. These results demonstrated a significant inhibitory effect of phosphite on PSF functionality. This inhibition likely stems from fundamental differences in the molecular structures of phosphite and phosphate, which influence their biochemical interactions. The observed suppression underscores the limited evolutionary adaptation of organisms to phosphite detoxification or metabolic assimilation. Consequently, phosphate persists as the dominant bioavailable phosphorus form on Earth. Finally, this induces its geological abundance and the lower metabolic cost for assimilation.

## Graphical Abstract

Schematic representation of the distinct effects of phosphate versus phosphite on *Aspergillus niger* and the proposed mechanisms mediating these responses. 
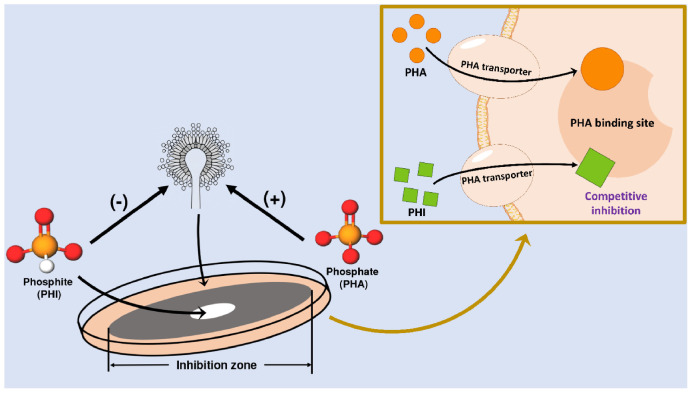

## 1 Introduction

Phosphorus (P) is a critical element for the metabolism of organisms (da Silva et al., 2023). While it predominantly exists as the +5 oxidation state in phosphate (PHA), P can also appear as other reduced forms, e.g., hypophosphite (+1), phosphite (PHI, +3) and hypophosphate (+4) (Morton and Edwards, 2005; Bindi et al., 2023). As a typical reduce P species, PHI is widely distributed in terrestrial and aquatic systems, including soils, freshwater, and marine environments (Hanrahan et al., 2005; Stone and White, 2012; Han et al., 2013; Pasek et al., 2014). Natural sources of PHI include meteorites, lightning strikes, volcanic eruption and microbial activity, whereas its anthropogenic sources are related to industrial production or use of relevant products (Morton and Edwards, 2005; Pasek et al., 2013; Liu et al., 2023). It is estimated that PHI concentration ranges from 0.1 to 1.3 μM in different environment, though levels may exceed 10 μM or even higher in some reduction conditions underground (Figueroa and Coates, 2017; Liu et al., 2023). PHI has been marketed for decades as the active ingredient released after hydrolysis of organic phosphonates and has emerged as an eco-friendly compound that bolsters plant resilience to abiotic and biotic stresses (Lambers et al., 2013; Koyukan et al., 2025).

The three-dimensional structure of PHI molecule is a balanced tetrahedron like PHA molecule, differing by the substitution of one oxygen atom with hydrogen (Tapia-Torres et al., 2016). Structural distinctions between PHI and PHA lead to significant variations in their solubility and ionic properties. PHI is about 1000 times more soluble than PHA in water (Figueroa and Coates, 2017). This enhanced solubility facilitates its practical use in agriculture, where PHI is typically administered to plants via aqueous solutions. Unlike PHA, which serves as a primary P fertilizer to boost crop yields, PHI is predominantly utilized as a fungistatic agent (Gómez-Merino and Trejo-Téllez, 2015; Achary et al., 2017). PHI combats phytopathogens by directly inhibiting fungal hyphal development and indirectly triggering systemic inducible resistance pathways in plants (Thao and Yamakawa, 2009; Mohammadi et al., 2021; Hunter et al., 2024). For instance, PHI treatment increased survival rates of papaya plants infected by *Phytophthora palmivora* to 93%, compared to 24% in untreated controls (Vawdrey and Westerhuis, 2007). PHI can inhibit phytopathogens particularly those belonging to the oomycetes (*Phytophthora* spp., *Pythium* spp.) (McDonald et al., 2001; Achary et al., 2017; Manghi et al., 2021). However, in the absence of PHA, where PHI is provided as the sole P source for plants, PHI usually shows negative effects on plant growth (Schroetter et al., 2006; Thao et al., 2008; Ratjen and Gerendás, 2009).

*Aspergillus niger* (*A. niger*) is one sizeable genus belonging to Aspergillaceae family. Owing to superior environmental adaptability and stress tolerance, *A. niger* is widespread across diverse environments (Yu et al., 2021). This fungus can produce various secondary metabolites, especially low molecular weight organic acids (Schneider et al., 2010). These metabolites acidify microenvironments to solubilize PHA minerals while simultaneously functioning as chelating agents to improve PO_4_^3–^ bioavailability (Khan et al., 2007; Klaic et al., 2021; Tian et al., 2021). Previous studies have demonstrated that *A. niger* can effectively leach heavy metals from contaminated soils or enhance plant uptake of heavy metals (Ren et al., 2009; Khan et al., 2019; Niu et al., 2021; Meng et al., 2024), which means *A. niger* plays a significant role in remediation of the contaminated soils. However, the influences of PHI on *A. niger* remain unexplored.

This study was aimed to characterize the response of *A. niger* to PHI exposure and quantitatively assess its effects. To comprehensively evaluate the influence of PHI, *A. niger* was cultured in both solid and liquid system.

## 2 Materials and methods

### 2.1 Fungal strain and chemicals

*Aspergillus niger* applied in this study has an accession number in China Center for Type Culture Collection (CCTCC) of M 2023240. The fungi were cultured on potato dextrose agar (PDA) at 28°C for 5 d to induce sporulation. After drenching the medium with sterile water, spores were scrapped from the surface and filtered through sterile gauze. The spore concentration was then determined and adjusted to 10^7^ cfu/mL with 0.85% sterile saline.

Sodium phosphite dibasic pentahydrate (Na_2_HPO_3_⋅5H_2_O, analytical reagent, Shanghai Macklin Biochemical, Ltd., China) was configured to different concentrations of solution. Sodium dihydrogen phosphate dihydrate (NaH_2_PO_4_⋅2H_2_O, analytical reagent, Nanjing Chemical Reagent, Ltd., China) was selected as PHA source for the disk-diffusion experiment and spectroscopical test.

### 2.2 Incubation experiment

The fungistatic effects of PHI and PHA were assessed using disk-diffusion, plate culture and liquid culture assays. In the disk-diffusion assay, fungal suspensions (100 μL) were inoculated onto PDA plates, followed by application of 6 mm sterile disks impregnated with 10 μL of 10^5^ μg/mL PHI or PHA solutions.

Six treatments were tested in plate cultures: Control (no PHI) and the solutions amended with PHI at 200, 400, 600, 800, or 1000 μg/mL (denoted as PHA@200PHI to PHA@1000PHI). The phosphate source in these solutions were originated from PDA. All treatments were performed in triplicate and incubated at 28°C for 5 d.

For liquid culture evaluation, PHI treatments were set consistent with the plate experiment (without agar). Spore suspensions (1 mL) were inoculated into the media and incubated at 28°C for 7 d with 180 rpm shaking. All the experiments were conducted in triplicate and all media were sterilized at 121°C for 20 min before experiments.

### 2.3 Chemical and microbial analyses

After 7 d of incubation, the liquid culture systems were filtered. The filtrate was prepared for pH and enzyme activity analysis. For the determination of biomass, the precipitate was carefully collected and subjected to a drying process at 65°C for 24 h in a controlled-temperature drying oven to ensure complete removal of residual moisture. After drying, the samples were weighed using an analytical balance with high precision to minimize measurement errors. The ACP (acid phosphatase) activity was analyzed by detection kit (ACP-1-W, Suzhou Keming Biotechnology Inc., China).

### 2.4 Instrumentation and data analyses

The growth of hypha and spores on agar media was observed by light microscope (LM, Olympus BX53, Japan). The pH value of liquid culture system was measured with an SG98 pH meter with an InLab Expert Pro-ISM-IP67 probe (Mettler Toledo Inc., USA). The data of ACP activity was recorded by a SpectraMax i3x Multi-Mode Microplate Reader (Molecular Devices, LLC., Austria) at 405 nm.

The attenuated total reflection fourier-transform infrared (ATR-IR) measurements were performed with a Thermo Scientific Nicolet iS5 Spectrometer (ThermoFisher Scientific Inc., USA) from 400 to 4000 cm^–1^. The recording was performed with 16 times scans for each sample at a spectral resolution of 4 cm^–1^. Raman spectra were obtained using Alpha 300 confocal Raman microscope (WITech, Germany). The spectral region of 100–4000 cm^–1^ was recorded using 473 nm laser.

One-way ANOVAs were used to test the datasets. Statistical significance was set at *p* < 0.05.

## 3 Results

### 3.1 Morphological evaluation of *A. niger*

**FIGURE 1:**
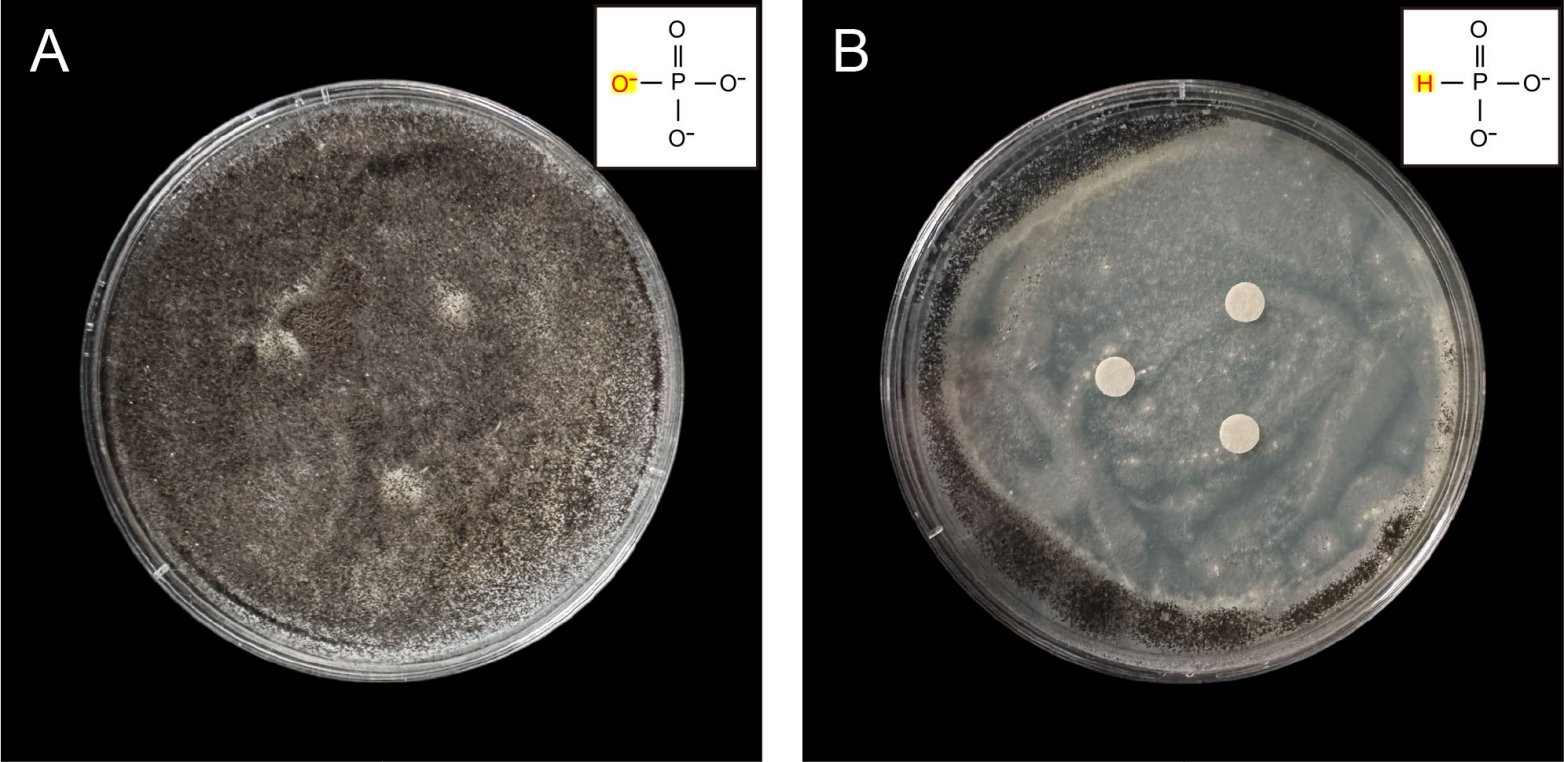
Antifungal activity by disk diffusion method of *A. niger* against PHI. The fungi suspension was inoculated onto plates using the spread plate method. **(A)** *A. niger* grown on PDA medium amended with 10^5^ μg/mL PHA, **(B)** *A. niger* grown on PDA medium amended with 10^5^ μg/mL PHI.

The growth of *A. niger* cultured with PHA formed a dense, dark brown mycelial network (Figure 1A). However, when PHI was added, *A. niger* exhibited restricted growth across the entire culture medium, with black spore production occurring exclusively at the colony periphery (Figure 1B). Notably, *A. niger* maintained growth despite the antifungal treatment, due to P availability in the PDA media.

**FIGURE 2:**
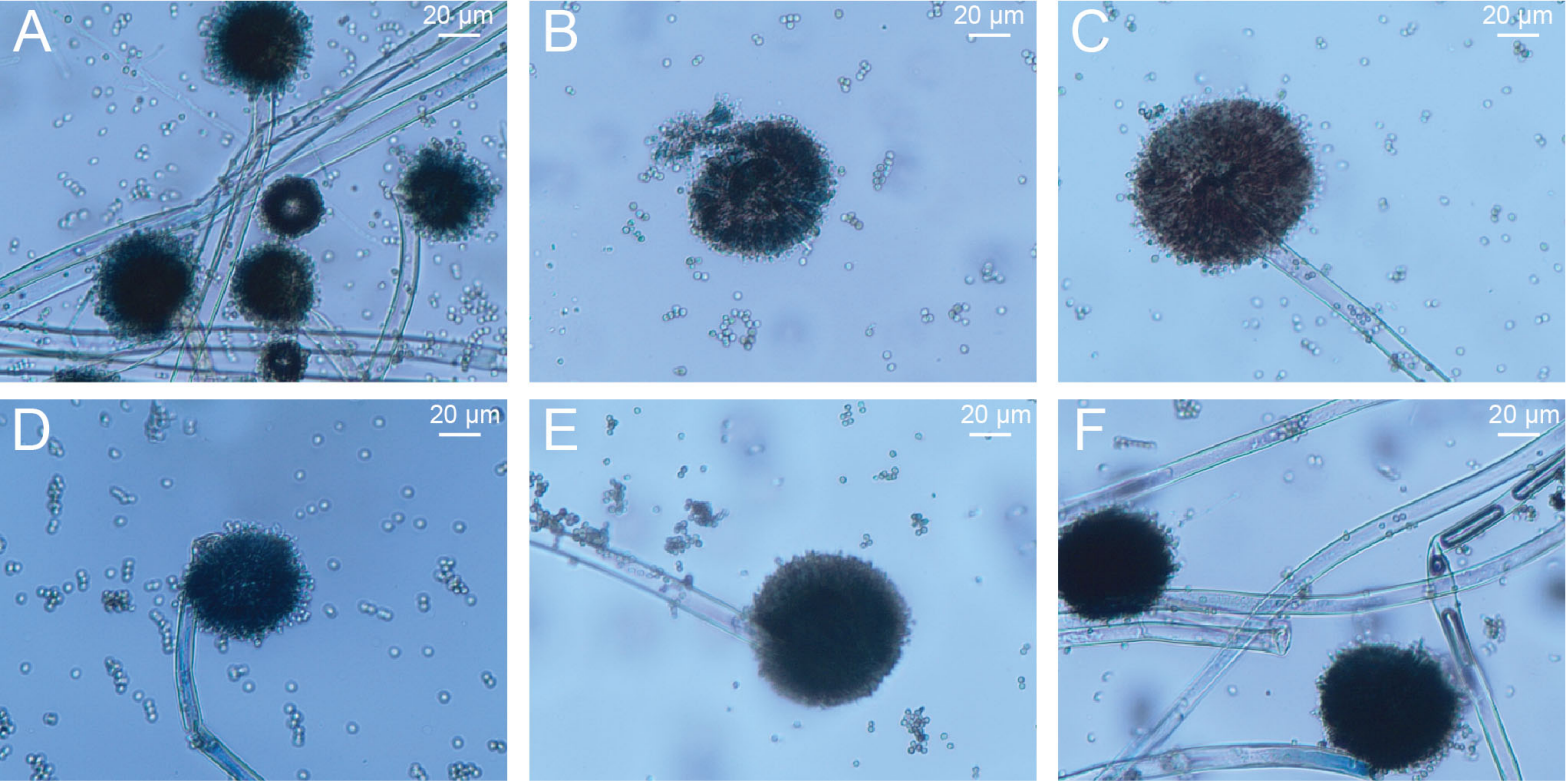
The growth of *A. niger* mycelia and spores on PDA solid culture media amended with different concentrations of PHI under light microscopy. **(A–F)** the corresponding concentrations of PHI were 0, 200, 400, 600, 800 and 1000 μg/mL.

Under LM, six distinct *A. niger* colonies could be observed in the PHI-free environment (Figure 2A). After PHI addition, the colony count decreased to one (Figures 2B–D). With increasing PHI concentration, we detected enhanced spore aggregation (Figure 2E). Figure 2F revealed a distinct colonial growth pattern characterized by sparse mycelial networks and compact spore clusters.

### 3.2 ATR-IR and Raman analysis

**FIGURE 3:**
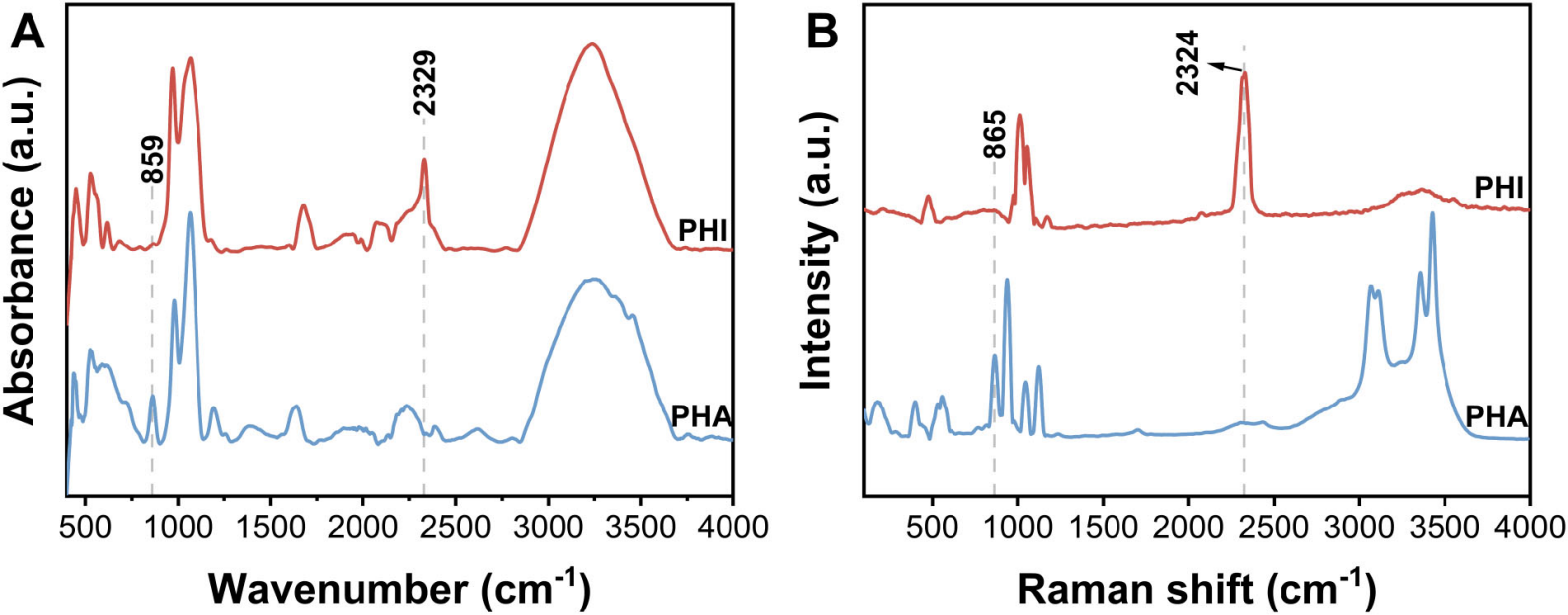
**(A)** ATR-IR and **(B)** Raman spectra of sodium phosphite dibasic pentahydrate (Na_2_HPO_3_⋅5H_2_O, PHI) and sodium dihydrogen phosphate dihydrate (NaH_2_PO_4_⋅2H_2_O, PHA). Data has been preprocessed with smoothing and baseline normalization.

PO_4_^3–^ ions can be mainly characterized by two types of vibrations, i.e., bending vibrations of O-P-O fragment (390-600 cm^–1^) and stretching vibrations of P-O (1000-1300 cm^–1^) (Ma et al., 2005; Jastrzêbski et al., 2011). In Figure 3, the ATR-IR and Raman spectra of PHA both contained the main bands originating from the above vibrations. The characteristic P-O-H stretching vibration observed at 860 cm^–1^ was served as a feature band for PHA in comparison with PHI samples (Kolandaivel et al., 1993; Oliveira et al., 2021).

Stretching vibrations and deformation modes of HPO_3_^2–^ anion are mainly divided to the following modes in IR spectrum, i.e., deformations of PO_3_ group (450-600 cm^–1^), stretching vibrations of PO_3_ group (967 cm^–1^), deformations of P-H (1031, 1063 cm^–1^), and stretching vibrations of P-H (2329 cm^–1^) (Chung et al., 2005; Ma et al., 2005; Kovrugin et al., 2017; Panicker, 2023). PHI exhibited coincident spectral bands in both Raman and IR across analogous wavenumber regions. Spectral analysis revealed that both PHA and PHI demonstrated overlapping vibrational features near 1000 cm^–1^, so the distinct band centered at ∼2300 cm^–1^ provided a diagnostic spectroscopic signature specific to PHI.

### 3.3 Physiological analysis

**FIGURE 4:**
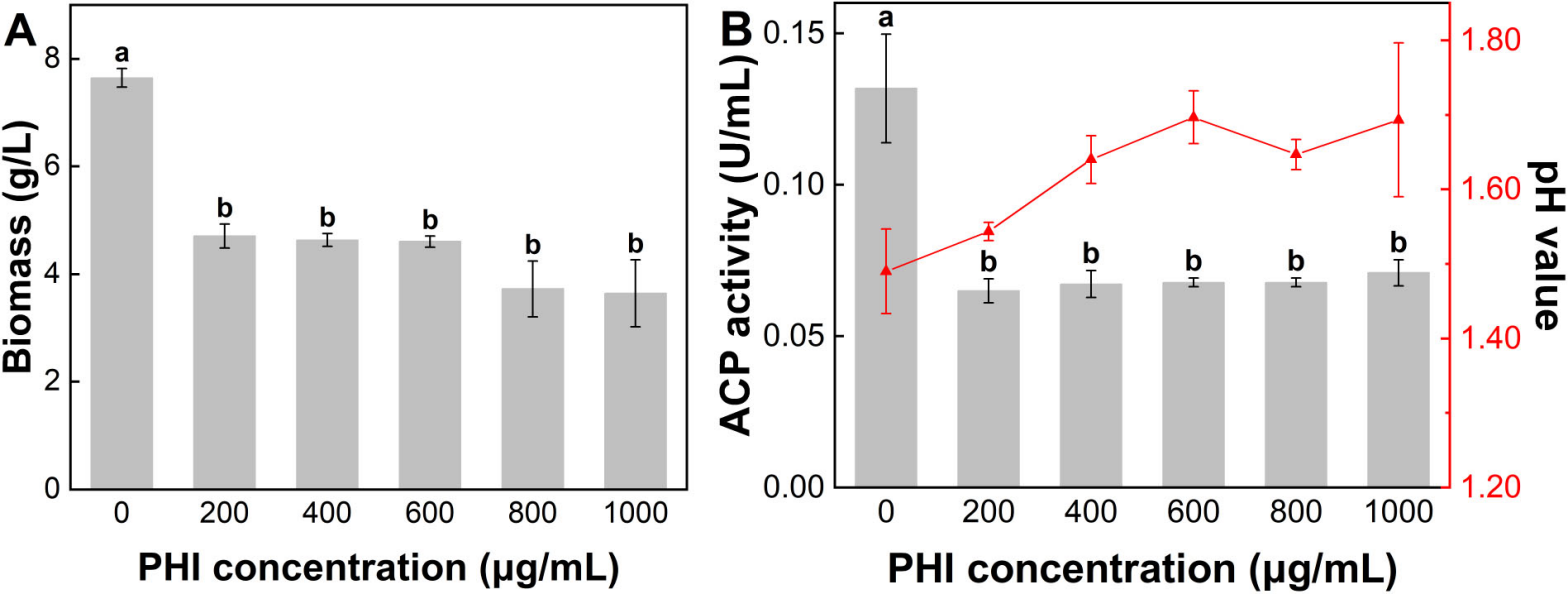
**(A)** The fungi biomass, **(B)** pH value and activity of acid phosphatase in the media after 7 d incubation. Lower-case letters above the bar indicate the significant differences among different PHI addition treatment (*p* < 0.05). Error bars represent the standard error.

The pH showed a significant increase with increasing PHI concentration (Figure 4B). The initial pH value of the media was 6.87, and initial pH of the system rose above 7 after adding PHI. After incubation, the pH of medium of control group was 1.49. In the treatment of PHA@200PHI, the pH slightly increased to 1.54. With increasing PHI concentration, PHA@800PHI and PHA@1000PHI, the pH value ultimately stabilized at approximately 1.65. These suggested that PHI inhibited the secretion of organic acids.

The microbial biomass measurements revealed significant variations among the experimental groups (Figure 4A). PHA group exhibited the highest biomass value at 7.65 g/L. Increasing the PHI concentration led to a gradual reduction in biomass. The biomass value of PHA@200PHI recorded 4.70 g/L. Slightly lower biomass value was observed in PHA@400PHI (4.63 g/L) and PHA@600PHI (4.60 g/L). Moreover, a decline occurred in PHA@800PHI (3.73 g/L) and PHA@1000PHI (3.65 g/L). Notably, the biomass of control group was approximately 79.58% higher than the average of the remaining groups (4.26 g/L).

ACP is a class of diverse enzymes catalyzing the P metabolism, which reflects the activity level of *A. niger*. In the treatment of PHA, *A. niger* exhibited the highest ACP activity (0.13 U/mL). A sharp decline in ACP activity occurred with the addition of PHI, with values stabilizing around 0.06–0.07 U/mL (Figure 4B). However, elevated PHI concentration did not appear to significantly enhance its inhibitory effect.

## 4 Discussion

PHI can significantly suppress mycelial growth of *A. niger*. Even in PHA@200PHI group, significant inhibition can be observed. However, the growth of *A. niger* was inhibited but not completely suppressed. In addition, there were no significant differences in ACP activity between PHI treatment group. These findings collectively support the point that PHI exhibits fungistatic rather than fungicidal properties.

As a substitute material for PHA, PHI has significant differences in its effect on *A. niger* compared with PHA. The distinct effects of PHI and PHA can be fundamentally attributed to differences in their molecular structures. Spectroscopic analysis was performed to identify structural fingerprints of PHA and PHI that may underlie their differential effects on microbial activity (Figure 3). PHI can be easily absorbed by organisms through PHA transporter due to their similarity (Jost et al., 2015; Achary et al., 2017). Nevertheless, PHI cannot participate in P metabolism in cells but affect normal P metabolism. Phosphorylases have binding sites to PHA anion, while PHI anion can compete with other ligands for the sites (Martin et al., 1998). PHI has only one face of the tetrahedron, relatively similar to all the faces of the PHA tetrahedron. When PHI binds to the enzyme, it is the P-H that protrudes from the enzyme surface, rather than P-O. Therefore, PHI is biologically incompatible with the metabolic processes mediated by PHA (McDonald et al., 2001). Previous studies have generally indicated this process, i.e., the inhibitory site of PHI on microorganisms is related to relevant enzymes in the metabolic pathways (Barchietto et al., 1992; Stehmann and Grant, 2000). For example, PHI may act directly on phosphoribosyl diphosphate (PRPP) synthase, which is an important intermediate in adenylate synthesis (Griffith et al., 1990; Hove-Jensen et al., 2016). PHI occupies the position of PHA, and the P metabolism cannot proceed normally. Eventually, the levels of ATP and NAD are decreased and the growth of the organisms is inhibited (Niere et al., 1990). In addition, PHI might also interfere with the function of the cytoskeleton and cell wall synthesis (King et al., 2010).

Existing evidences indicate that prebiotic P geochemistry was dominated by reduced oxidation-state compounds, particularly PHI (Gulick, 1957; Li et al., 2025; Pasek, 2008). Some organisms from anoxic marine mud can perform DPO (dissimilatory phosphite oxidation) pathway, which means that they can utilize HPO_3_^2–^ as the sole electron donor and energy source, coupling its oxidation to cellular growth and replication (Walton et al., 2023). However, PHA exhibits higher thermodynamic stability under oxidizing conditions (Pasek and Lauretta, 2005). Following planetary oxygenation and biological diversification, PHI underwent progressive oxidation to PHA. Thus, this redox transition establishes the contemporary dominance of PHA in terrestrial P pools. This preference is also reflected in biomineralized tissues such as bones and teeth, which incorporate P exclusively in its +5 oxidation state. It is notable that PHI assimilation pathways are absent in most extant organisms (Loera-Quezada et al., 2015), potentially explaining its cytotoxic effects when it serves as the only P source for organisms. Many reduced P cycling genes are only expressed in microbial communities when more bioavailable forms of P limited (Boden et al., 2024). Microbial utilization of PHA as a P source is favored by their environmental abundance, chemical stability, and the lower energy demand. This evolutionary selection reflects an optimization for P metabolic efficiency, leaving PHI as a marginal relic in modern ecosystems.

## 5 Conclusion

The results demonstrate that PHI exerts significant inhibitory effects on the growth and sporulation of *A. niger*, a model phosphorus-solubilizing fungus. This finding suggests potential applications of alternative phosphate materials in fungal control strategies. However, the potential for efficiently utilization of PHI requires further investigation. Subsequent research should evaluate the dynamic responses to better understand the long-term efficacy of PHI as an inhibitory agent to functional fungi.

## Data Availability

The raw data supporting the conclusions of this article will be made available by the authors, without undue reservation.
